# Supplementary figures and images for: Mitochondrial regulator PGC-1a in neuronal metabolism and brain aging
Source: bioRxiv. 2023 Sep 29:2023.09.29.559526. Preprint. [Version 1] doi: 10.1101/2023.09.29.559526 (PMC10557769; doi:10.1101/2023.09.29.559526)

**A**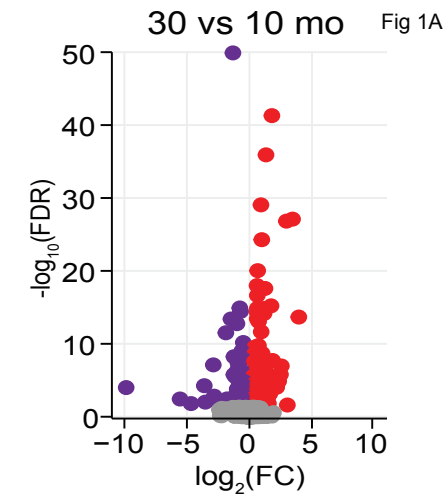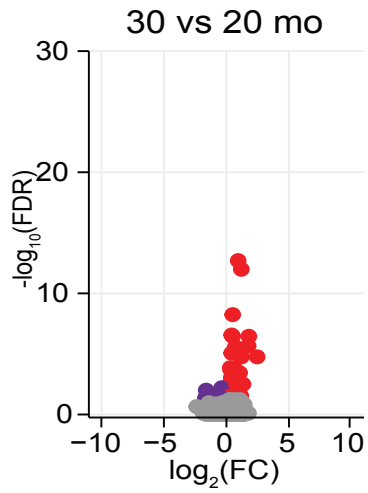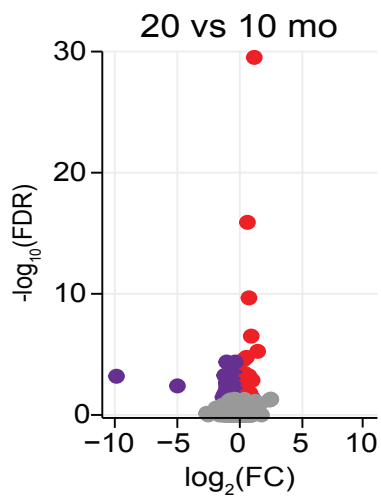**B**

30 v 10

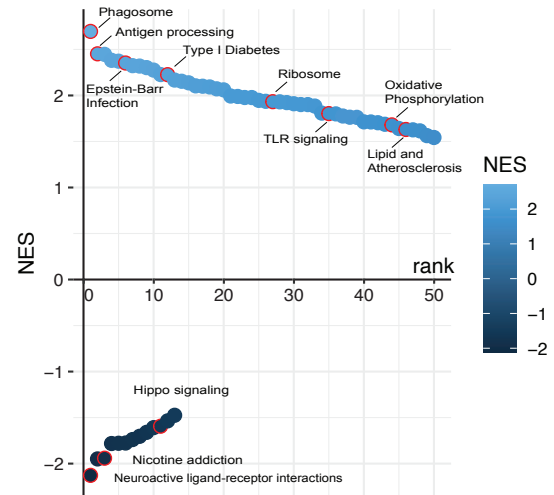

30 v 20

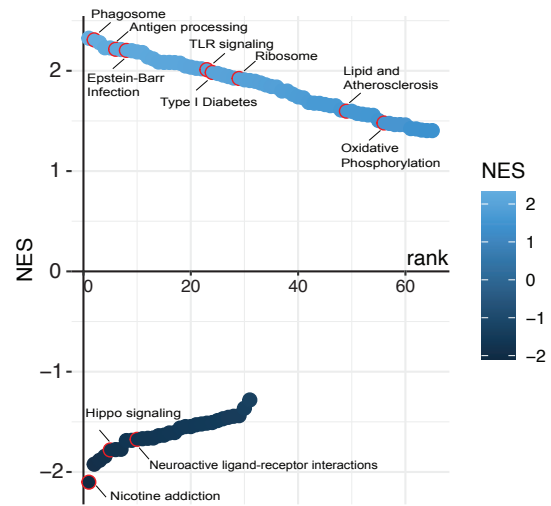

20 v 10

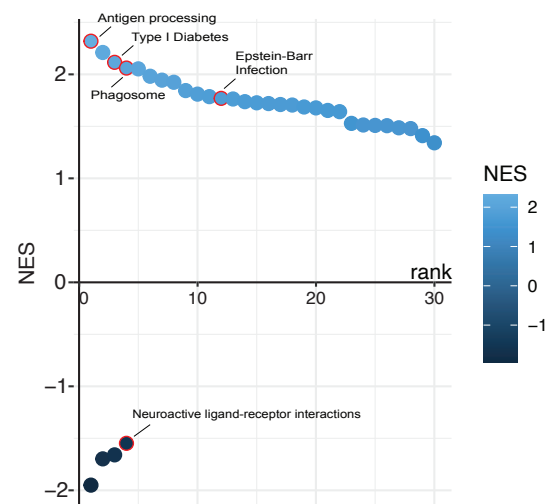

Supplement: Supplement 2 [file media-2.pdf]

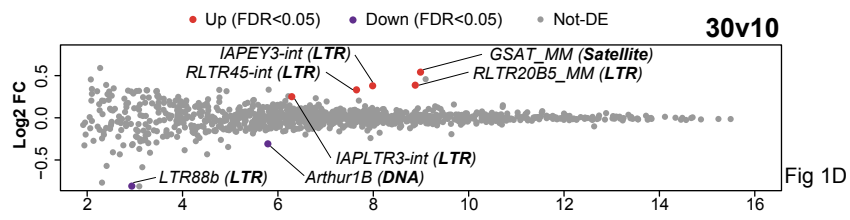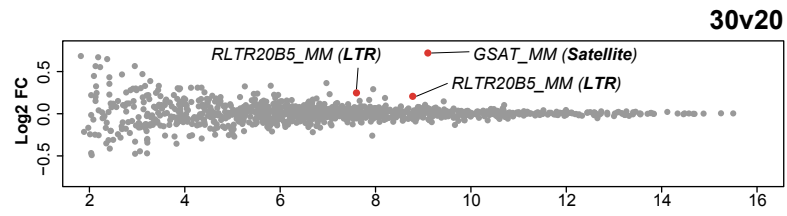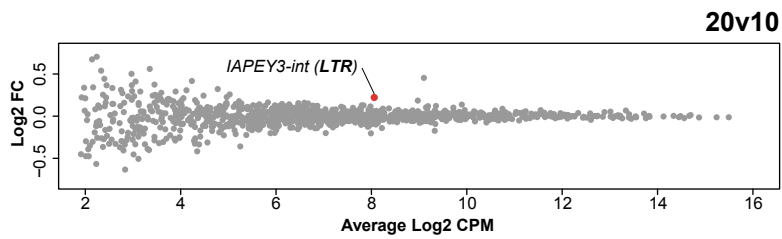

Supplement: Supplement 3 [file media-3.pdf]

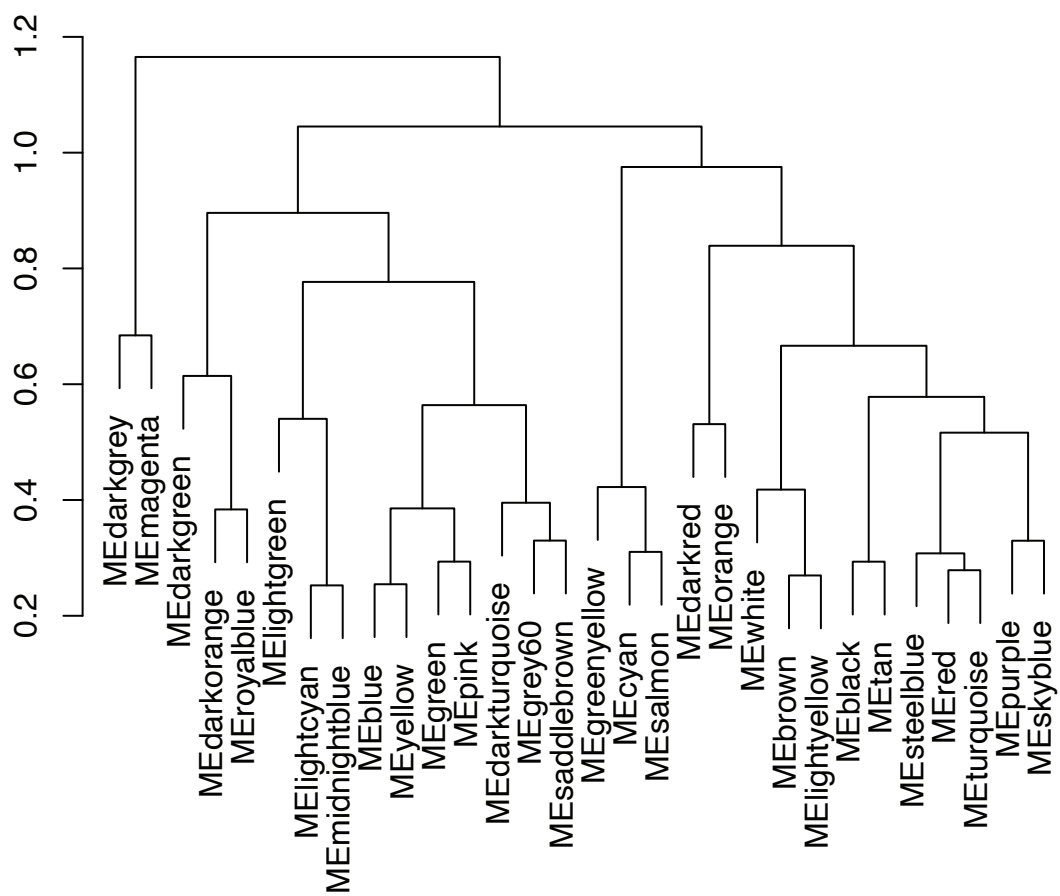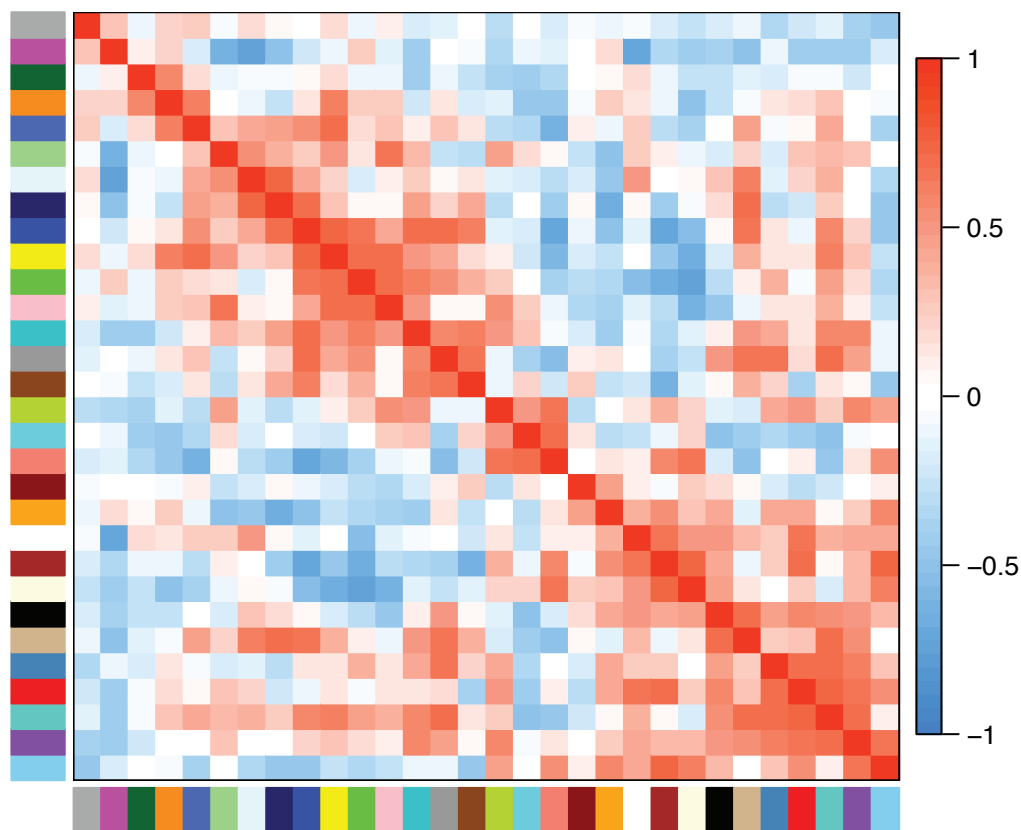

Supplement: Supplement 4 [file media-4.pdf]

## NSC Differentiation

PGC1a1

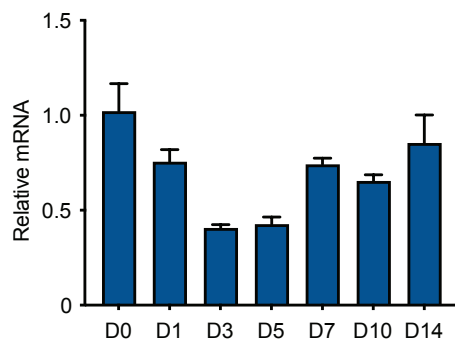

PGC1a4

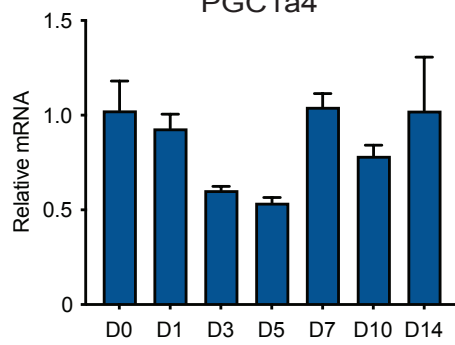

Supplement: Supplement 5 [file media-5.pdf]

Supplemental Figure: Neuron v. Astrocyte: MPLSM-FLIM Analysis

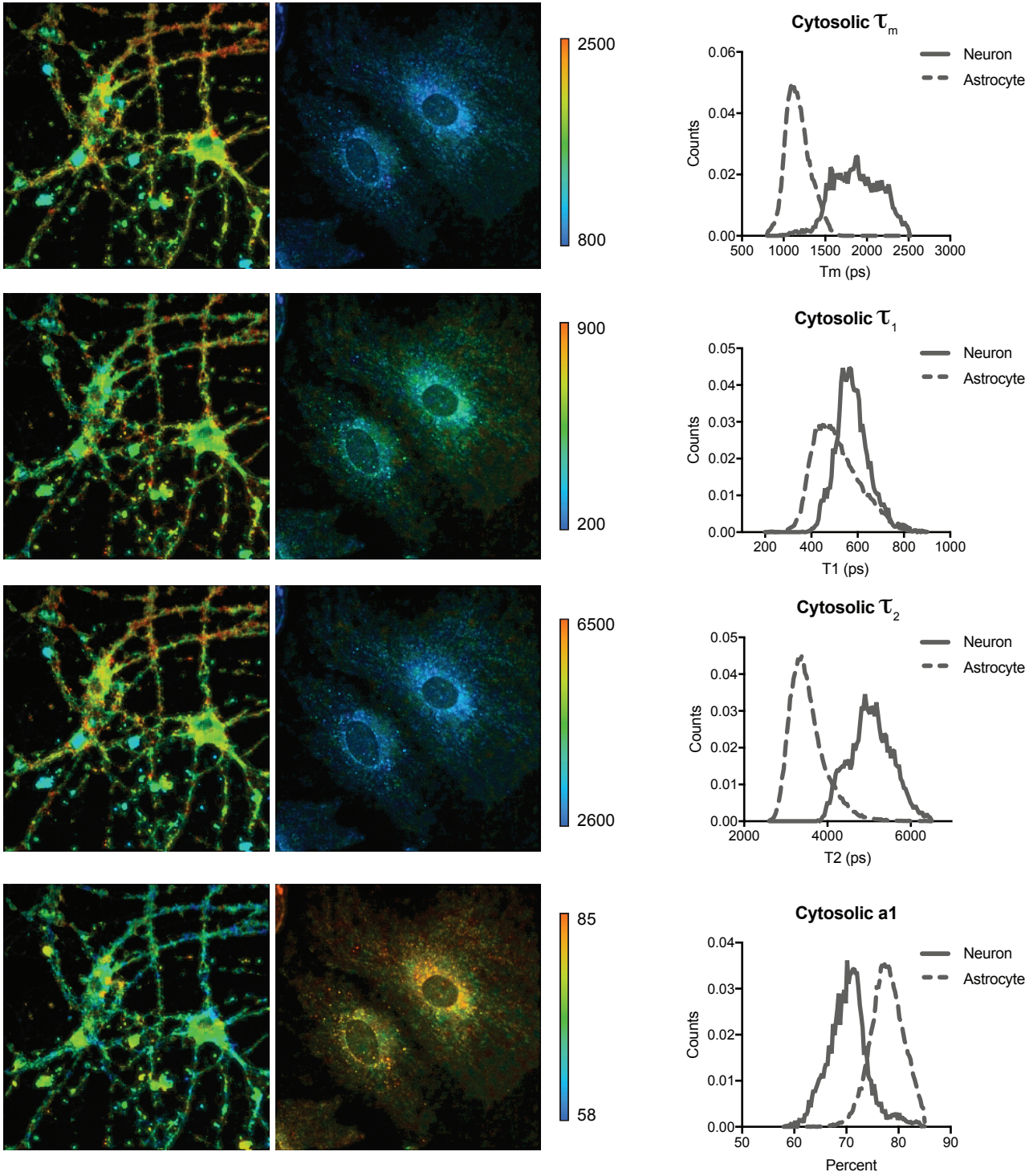

Supplement: Supplement 6 [file media-6.pdf]

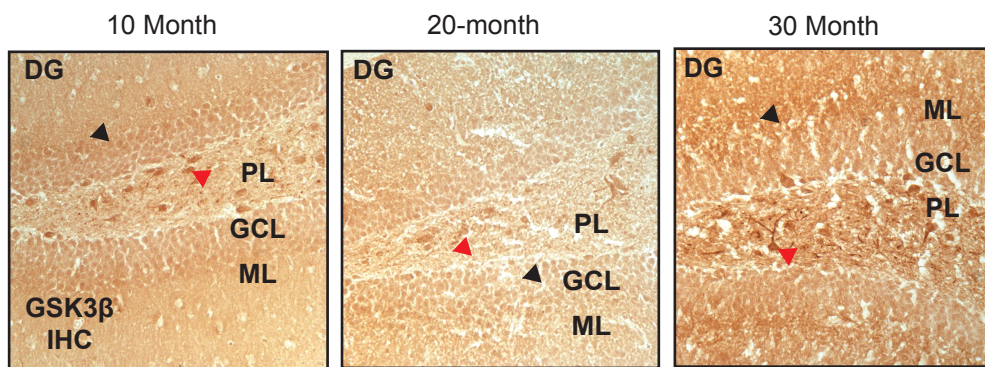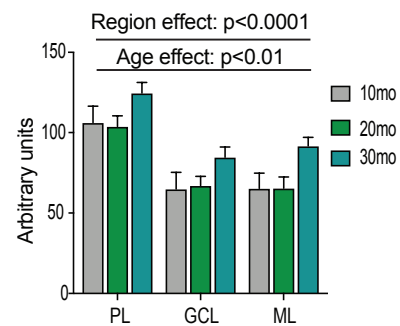

Supplement: Supplement 7 [file media-7.pdf]

# P1 Astrocytes

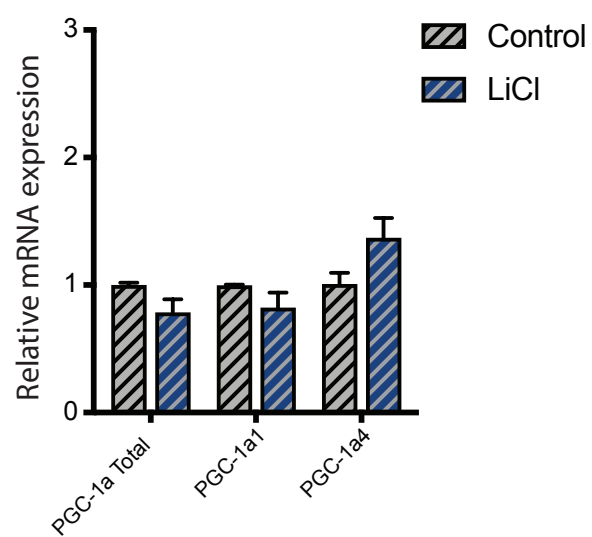

Supplement: Supplement 8 [file media-8.pdf]
