# Supplementary material for: Mitochondrial regulator PGC-1a in neuronal metabolism and brain aging
Source: bioRxiv. 2023 Sep 29:2023.09.29.559526. Preprint. [Version 1] doi: 10.1101/2023.09.29.559526 (PMC10557769; doi:10.1101/2023.09.29.559526)
Supplement: Supplement 9 [file media-9.docx]

Supplementary Table 1: Differentially expressed genes and enriched KEGG pathways detected by GSEA of aged mouse brains.

Supplementary Table 2: Modules produced by WGCNA of aged mouse brains.

Supplementary Table 3: RT-qPCR primer sequences for PGC-1a transcript variants.

Supplementary Table 4: Differentially expressed proteins and significantly enriched KEGG pathways detected by overrepresentation analysis in aged mouse brains.

Supplementary Table 5: Differentially expressed genes and significantly enriched pathways detected by gene set enrichment analysis in LiCl-treated neurons.

Supplementary Table 6: Differentially expressed genes and significantly enriched pathways detected in brains of LiCO3-fed mice.
